# Middle East respiratory syndrome coronavirus ORF4b protein inhibits type I interferon production through both cytoplasmic and nuclear targets

**DOI:** 10.1038/srep17554

**Published:** 2015-12-03

**Authors:** Yang Yang, Fei Ye, Na Zhu, Wenling Wang, Yao Deng, Zhengdong Zhao, Wenjie Tan

**Affiliations:** 1 Key Laboratory of Medical Virology, Ministry of Health; National Institute for Viral Disease Control and Prevention, Chinese Center for Disease Control and Prevention, Beijing, 102206, China; 2 Shenzhen Key Laboratory of Pathogen and Immunity, Shenzhen Third People’s Hospital, Shenzhen, China; 3 Key Laboratory of Pathogen System Biology, Ministry of Health; Institute of Pathogen Biology, Chinese Academy of Medical Sciences, Beijing, 100176, China

## Abstract

Middle East respiratory syndrome coronavirus (MERS-CoV) is a novel and highly pathogenic human coronavirus and has quickly spread to other countries in the Middle East, Europe, North Africa and Asia since 2012. Previous studies have shown that MERS-CoV ORF4b antagonizes the early antiviral alpha/beta interferon (IFN-α/β) response, which may significantly contribute to MERS-CoV pathogenesis; however, the underlying mechanism is poorly understood. Here, we found that ORF4b in the cytoplasm could specifically bind to TANK binding kinase 1 (TBK1) and IκB kinase epsilon (IKKε), suppress the molecular interaction between mitochondrial antiviral signaling protein (MAVS) and IKKε, and inhibit IFN regulatory factor 3 (IRF3) phosphorylation and subsequent IFN-β production. Further analysis showed that ORF4b could also inhibit IRF3 and IRF7-induced production of IFN-β, whereas deletion of the nuclear localization signal of ORF4b abrogated its ability to inhibit IRF3 and IRF7-induced production of IFN-β, but not IFN-β production induced by RIG-I, MDA5, MAVS, IKKε, and TBK-1, suggesting that ORF4b could inhibit the induction of IFN-β in both the cytoplasm and nucleus. Collectively, these results indicate that MERS-CoV ORF4b inhibits the induction of type I IFN through a direct interaction with IKKε/TBK1 in the cytoplasm, and also in the nucleus with unknown mechanism. Viruses have evolved multiple strategies to evade or thwart a host’s antiviral responses. A novel human coronavirus (HCoV), Middle East respiratory syndrome coronavirus (MERS-CoV), is distinguished from other coronaviruses by its high pathogenicity and mortality. However, virulence determinants that distinguish MERS-CoV from other HCoVs have yet to be identified. MERS-CoV ORF4b antagonizes the early antiviral response, which may contribute to MERS-CoV pathogenesis. Here, we report the identification of the interferon (IFN) antagonism mechanism of MERS-CoV ORF4b. MERS-CoV ORF4b inhibits the production of type I IFN through a direct interaction with IKKε/TBK1 in the cytoplasm, and also in the nucleus with unknown mechanism. These findings provide a rationale for the novel pathogenesis of MERS-CoV as well as a basis for developing a candidate therapeutic against this virus.

Middle East respiratory syndrome coronavirus (MERS-CoV) is a novel and highly pathogenic human coronavirus that emerged in Jeddah of Saudi Arabia and has rapidly spread to other countries in the Middle East, Europe, and North Africa since 2012^1^,^2^. As of October 12, 2015, the World Health Organization has been notified of 1,595 laboratory-confirmed cases of infection with MERS-CoV, including 571 related deaths^2^. The identification of clusters of coronavirus-infected cases, especially the recent outbreak in South Korea, indicates that MERS-CoV can be transmitted from human-to-human^3^,^4^,^5^, and raises concern regarding a possible outbreak similar to the one caused by the severe acute respiratory syndrome-related coronavirus (SARS-CoV) in 2002–2003^6^,^7^.

An important hallmark of virulence is the extent to which viruses are able to cope with the antiviral type I IFN system, which is a major part of the innate immune response^8^,^9^. Similar to SARS-CoV, MERS-CoV can cause a lethal infectious disease in humans, characterized by an aberrant immune response^10^. Previous studies have shown that MERS-CoV infection failed to elicit a strong type I or III IFN or proinflammatory innate immune responses in *ex vivo* respiratory tissue cultures^11^,^12^, and its replication was susceptible to IFN alpha^13^,^14^. Until recently, this inhibition was thought to be mediated through viral structural, accessory, and nonstructural proteins M, ORF4a, ORF4b, ORF5, and papain-like protease (PLpro)^15^,^16^,^17^,^18^,^19^,^20^.

IFN and IFN-induced cellular antiviral responses are the first line of defense against viral infection. Mammalian hosts have evolved a variety of cellular sensors for viral infection known as pattern recognition receptors (PRRs), and it is the engagement of these protein receptors that ultimately leads to the production of type I IFN through complex and redundant pathways^8^,^9^. Retinoic acid-induced gene I (RIG-I) and melanoma differentiation gene 5 (MDA5) are important cytoplasmic PRRs that recognize viral RNAs^21^. Upon sensing cytoplasmic viral RNAs, RIG-I and MDA5 associate with the mitochondrial signaling adapter MAVS (also known as IPS-1/Cardif/VISA)^22^,^23^,^24^, which subsequently recruits TBK1 and IKKε kinases. Activation of TBK1 and IKKε results in the phosphorylation of IRF-3 or IRF-7, translocation to the nucleus, and induction of IFN-β mRNA transcription and translation.

Among the five IFN antagonist proteins of MERS-CoV, only the underlying mechanism of ORF4a has been characterized^18^. ORF4a protein interacts with PACT in an RNA-dependent manner and inhibits PACT-induced activation of RIG-I and MDA5. ORF4b is another important antagonist viral protein of MERS-CoV, and inhibits SeV-induced IRF3 translocation and subsequent IFN-β production. It is constantly expressed during MERS-CoV infection and predominantly localizes to the nucleus with a small part dispersed throughout the cytoplasm^15^,^17^. Reverse genetics have shown that ORF4b is not required for viral replication, whereas MERS-ΔORF3–5 was reduced by 1–1.5 logs compared to rMERS-CoV^25^,^26^.

In the present study, we investigated the molecular mechanisms by which ORF4b protein inhibits IFN signaling. We showed that ORF4b specifically binds to TBK1 and IKKε, suppresses the molecular interaction between MAVS and IKKε, and inhibits IRF3 phosphorylation and subsequent IFN-β production. Interestingly, ORF4b also inhibited IRF3 and IRF7-induced production of IFN-β, whereas deletion of the nuclear localization signal of ORF4b limited its ability to inhibit IRF3 and IRF7-induced production of IFN-β, but not the IFN-β production induced by RIG-I, MDA5, MAVS, IKKε, and TBK-1. These data suggest that ORF4b inhibits the induction of IFN-β in both the cytoplasm and nucleus.

## Results

### ORF4b inhibits induction of IFN-β in a dose-dependent manner, but does not induce apoptosis

**Figure 1:**
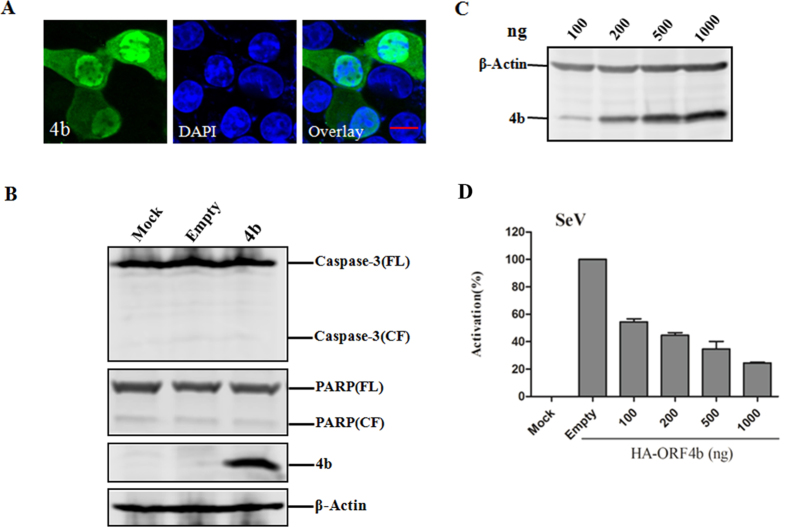
ORF4b predominantly localizes to the nucleolus and inhibits the induction of the IFN-β in a dose-dependent manner. (**A**) Nuclear localization of ORF4b protein. HeLa cells were transfected with an expression plasmid for HA-tagged ORF4b protein, and then stained for ORF4b with anti-HA antibody (green) at 24 h post-transfection. Nuclei were stained with DAPI (blue). Cells were analyzed by confocal microscopy using a 100× objective, and representative images are shown. Scale bar, 20 μm. (**B**) ORF4b could not induce apoptosis. HeLa cells were transfected with an expression plasmid for HA-tagged ORF4b protein, and cell lysates were analyzed by Western blotting with anti-Caspase-3 and anti-PARP antibodies. FL indicates full-length and CF indicates cleaved fragment. (**C**) expression of ORF4b protein in cultured cells. The 293T cells were transfected with increasing amounts of an expression plasmid for HA-tagged ORF4b protein, and cell lysates were analyzed by Western blotting. (**D**) ORF4b inhibits the induction of IFN-β in a dose-dependent manner. 293T cells were co-transfected with pGL3-IFNβ-luc, the internal control pRL-SV40, and increasing amounts of plasmid expressing ORF4b. At 24 h post-transfection, cells were infected with Sendai virus, then cells were harvested at 24 h post-infection and analyzed for Firefly and Renilla luciferase. Data are representative of three independent experiments with triplicate samples.

It was previously reported that ORF4b mainly co-localized with the nucleus and functions as an IFN antagonist^15^,^17^; however, the underlying mechanism remains unknown. Thus, we characterized ORF4b-mediated inhibition of type I IFN production. First, a plasmid encoding ORF4b was transfected into HeLa cells, and the subcellular localization of ORF4b was observed. Consistent with previous reports, ORF4b protein predominantly localized to the nucleus with a fraction dispersed throughout the cytoplasm (Fig. 1A). Furthermore, unlike SARS ORF3b^27^, ORF4b-expressing cells did not show growth defects or signs of apoptosis (Fig. 1B). To confirm the inhibitory function of type I IFN production, we transfected 293T cells with increasing amounts of ORF4b-expressing plasmid (Fig. 1C), together with a plasmid expressing firefly luciferase reporter driven by the IFN-β promoter and a plasmid expressing Renilla luciferase that served as the internal control. As expected, expression of ORF4b protein resulted in the significant suppression of SeV-induced activation of the IFN promoter in a dose-dependent manner (Fig. 1D).

### ORF4b inhibits IFN-β expression by targeting MDA5, TBK1, and IKKε

**Figure 2:**
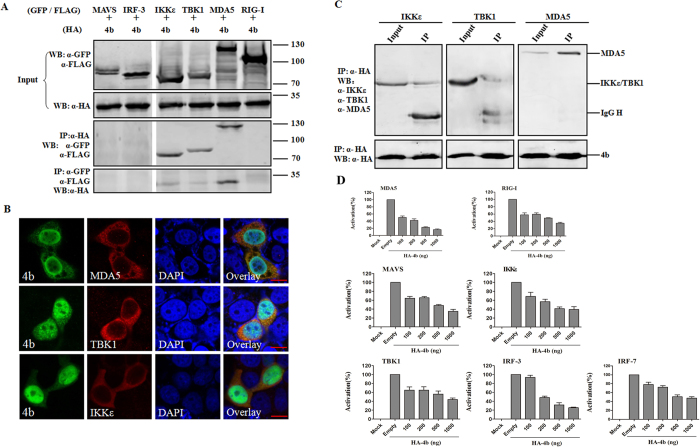
ORF4b can inhibit IFN-β expression by targeting MDA5, TBK1, and IKKε. **(A**) Association of ORF4b protein with MDA5, TBK1, and IKKε. The 293T cells were co-transfected with expression plasmids for HA-tagged ORF4b protein and the indicated transducer proteins including GFP-tagged MAVS and IRF3, FLAG-tagged IKKε, TBK1, MDA5, and RIG-I. Input cell lysates and immunoprecipitates were analyzed by Western blotting (WB) with anti-GFP (α-GFP), anti-FLAG (α-FLAG), and anti-HA (α-HA) antibodies. (**B**) Co-localization of ORF4b protein with MDA5, TBK1, and IKKε. HeLa cells were co-transfected with expression plasmids for HA-tagged ORF4b protein and the indicated expression plasmids for Flag-tagged MDA5, TBK1, and IKKε. Cells were then stained for ORF4b and MDA5/TBK1/IKKε with anti-HA and anti-FLAG antibodies, respectively. The green (ORF4b) and red (MDA5/TBK1/IKKε) fluorescent signals were merged. Nuclei were stained with DAPI (blue). Cells were analyzed by confocal microscopy using a 100× objective, and representative images are shown. Scale bar, 20 μm. (**C**) association of ORF4b protein with endogenous MDA5, TBK1, and IKKε. 293T cells were transfected with ORF4b expressing plasmid and immunoprecipitated by anti-HA monoclonal antibody, and the co-immunoprecipitation was assessed by Western blotting using anti-MDA5, IKKε, and TBK1 monoclonal antibody. (**D**) ORF4b could also inhibit interferon-inducing activity of IRF-3 and IRF7. Experiments were carried out as in Fig. 1D except that 293T cells were not stimulated with SeV but co-transfected with plasmids expressing MDA5, RIG-I, MAVS, TBK1, IKKε, IRF3 and IRF7. Data are representative of three independent experiments with triplicate samples.

SeV is a strong inducer of the RIG-I-like receptor (RLR)-mediated IFN signaling pathway^23^. ORF4b-mediated inhibition of SeV-induced IFN-β production and IRF3 activation suggests that the protein targets one or several components of the RLR signaling pathway. RIG-I and MDA5 recognize 5′-triphosphate RNA and dsRNA from RNA viruses and initiate host antiviral responses, whereby a number of downstream molecules are recruited or activated^21^. To characterize the possible step and molecular target of ORF4b in the IFN induction signaling pathway, reciprocal co-immunoprecipitation (co-IP) experiments were performed. The 293T cells were transfected with expression plasmids for Flag-tagged RIG-I, MDA5, IKKε, TBK1, and GFP-tagged IRF3 and MAVS with ORF4b. Although comparable expression levels of both ORF4b protein and the transducer proteins were observed in 293T cells, ORF4b protein co-immunoprecipitated with MDA5, IKKε, and TBK1, but not with RIG-I, MAVS, or IRF3 (Fig. 2A). Notably, precipitation with HA or Flag/GFP antibodies yielded similar results (Fig. 2A), and also the co-localization of ORF4b and MDA5, IKKε, and TBK1 was found in HeLa cells (Fig. 2B), suggesting that the ORF4b protein interacts with MDA5, IKKε, and TBK1. Furthermore, to determine whether ORF4b interacts with the endogenous MDA5, IKKε, and TBK1, we transfected 293T cells with ORF4b expressing plasmid. Then ORF4b was immunoprecipitated by anti-HA monoclonal antibody, and the co-immunoprecipitation was assessed by Western blotting using anti-MDA5, IKKε, and TBK1 monoclonal antibody. In agreement with the former observation, the endogenous MDA5, IKKε, and TBK1 proteins were successfully co-immunoprecipitated (Fig. 2C). These results suggest that ORF4b inhibited IFN-β expression by targeting MDA5, TBK1, and IKKε, a step upstream of IRF3.

To further confirm the inhibition step of ORF4b, we transfected plasmid expressing HA-tagged ORF4b and plasmids expressing various transducer proteins that stimulate IFN production in the RIG-I signaling pathway, including RIG-I, MDA5, MAVS, IKKε, TBK1, IRF3 and IRF7, into 293T cells together with the IFN-β reporter plasmid and the internal control. Interestingly, ORF4b inhibited the induction of IFN-β by RIG-I, MDA5, MAVS, IKKε, and TBK-1 (Fig. 2D). However, consistent with previous reports^17^, ORF4b also inhibited IRF3 and IRF7-induced production of IFN-β (Fig. 2D), suggesting that ORF4b may function in the nucleus to inhibit type I IFN production.

### Deletion of the nuclear localization signal in ORF4b inhibits the induction of IFN-β, but is unable to inhibit IRF3 and IRF7-induced production of IFN-β

**Figure 3:**
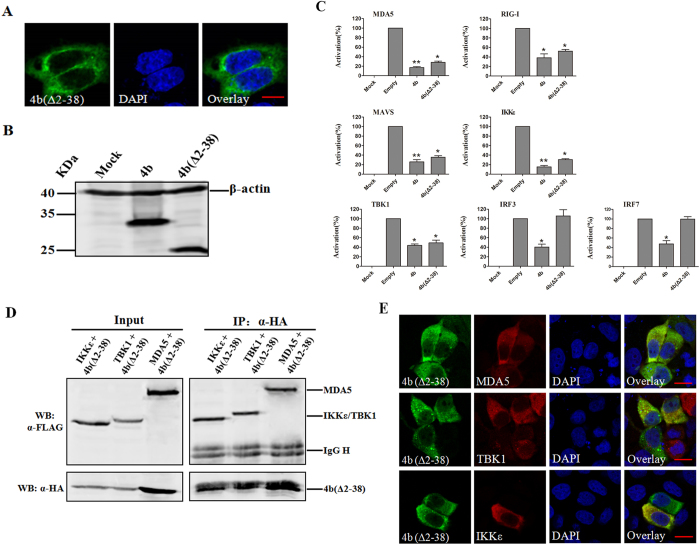
4b(Δ2-38) localizes to the cytoplasm and cannot inhibit IRF3 induced production of IFN-β. (**A**) 4b(Δ2-38) localizes to the cytoplasm. Scale bar, 10 μm. (**B**) expression of ORF4b and ORF4b(Δ2-38) proteins in cultured cells. (**C**) 4b(Δ2-38) inhibits interferon-inducing activity of RIG-I/MDA5, MAVS, and TBK1/IKKε, but not IRF-3 and IRF7. Data are representative of three independent experiments with triplicate samples. *P < 0.05; **P < 0.01 versus empty (Students’ t-test). (**D**) 4b(Δ2-38) interacts with MDA5, IKKε and TBK1. (**E**) co-localization of 4b(Δ2-38) protein with MDA5, TBK1, and IKKε. Scale bar, 20 μm. Experiments were carried out as in Figs 1A,C and 2A,B,D respectively, except that 4b(Δ2-38) was accessed.

Since ORF4b localizes to the nucleus, which may contribute to inhibition of IRF3-induced production of IFN-β, it implied that ORF4b could also inhibit the induction of IFN-β in the nucleus. We hypothesized that abolishing the nuclear import of ORF4b would result in its inability to inhibit IRF3-induced production of IFN-β. To explore this hypothesis, we constructed a truncated ORF4b with a deletion of the N-terminal 2–38 amino acids (aa), which correspond to the predicted NLS-containing region. As expected, ORF4b (Δ2–38) exclusively localized to the cytoplasm (Fig. 3A), and inhibited the induction of IFN-β by RIG-I, MDA5, MAVS, IKKε, and TBK-1 at comparable levels as ORF4b, but not IRF3 and IRF7 (Fig. 3B,C). In accordance with these results, ORF4b (Δ2–38) was easily found to interact and co-localize with MDA5, IKKε, and TBK-1 (Fig. 3D,E). These data indicate that ORF4b can inhibit the induction of IFN-β in both the cytoplasm and nucleus.

### ORF4b protein does not affect formation of the complex between MDA5 and MAVS

**Figure 4:**
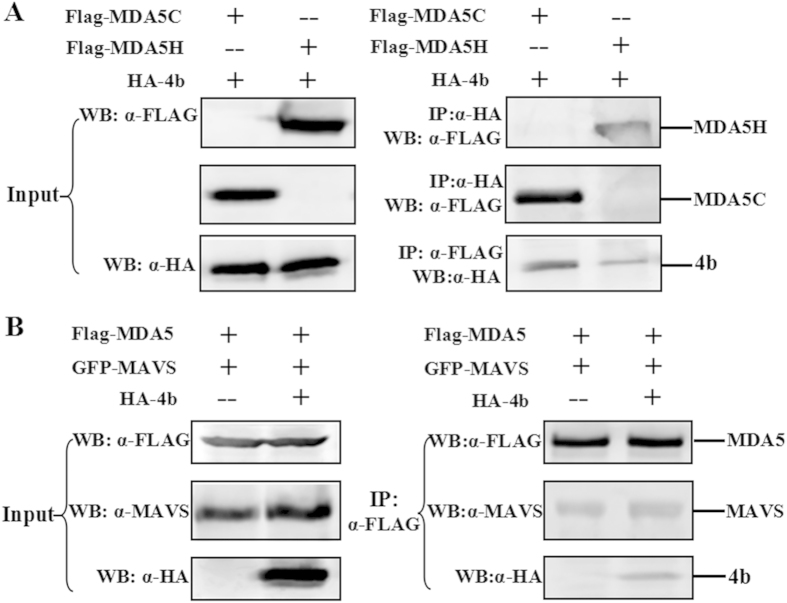
ORF4b has a stronger interaction with the amino terminus of MDA5, and does not disrupt formation of the complex between MDA-5 and MAVS. (**A**) ORF4b protein mainly interacts with the amino terminus MDA5. The 293T cells were co-transfected with expression plasmids for HA-tagged ORF4b protein, and the indicated expression plasmids for MDA5C containing the amino-terminal domain (aa 1-287) or MDA5H containing the carboxyl-terminal domain (aa 287-1025). Input cell lysates (left panel) and immunoprecipitates (right panel) were analyzed by Western blotting (WB) with anti-FLAG (α-FLAG) and anti-HA (α-HA) antibodies. (**B**) ORF4b does not disrupt the formation of complex between MDA5 and MAVS. The 293T cells were co-transfected with expression plasmids for FLAG-tagged MDA5 and GFP-tagged MAVS and plasmid expressing HA-tagged ORF4b or empty vector. Input cell lysates (left panel) and immunoprecipitates (right panel) were analyzed by Western blotting (WB) with anti-MAVS (α-MAVS), anti-FLAG (α-FLAG), and anti-HA (α-HA) antibodies.

MDA5 is one of the most important cytoplasmic PRRs that recognize viral RNAs^21^. Upon sensing cytoplasmic viral RNAs, MDA5 associates with the mitochondrial signaling adapter MAVS through its CARD domain and activates IFN signaling^22^,^23^,^24^. To determine which region of MDA-5 binds ORF4b, MDA-5 mutants containing either the amino-terminal CARD domain (aa 1–287; Flag-MDA-5C) or the carboxyl terminal helicase domain (aa 287–1025; Flag-MDA-5H) were tested^21^,^28^. In 293T cells co-transfected with ORF4b and either Flag-MDA5C or Flag-MDA5H, ORF4b co-immunoprecipitated with both constructs, whereas a stronger interaction was observed with MDA5C (Fig. 4A). Because MDA5 recognizes viral RNA, it delivers the activation signal to MAVS through a direct interaction via its CARD motif. Therefore, we explored whether ORF4b protein impaired this process. The 293T cells were co-transfected with MDA5 and MAVS, along with ORF4b-expressing plasmid or an empty vector. MDA5 was immunoprecipitated by anti-Flag monoclonal antibody, and the co-immunoprecipitation of MAVS was assessed by Western blotting using anti-MAVS monoclonal antibody. As expected, MDA5 co-immunoprecipitated with MAVS, with no difference in the absence or presence of ORF4b (Fig. 4B).

### IRF3 phosphorylation and nuclear translocation are inhibited by ORF4b protein

**Figure 5:**
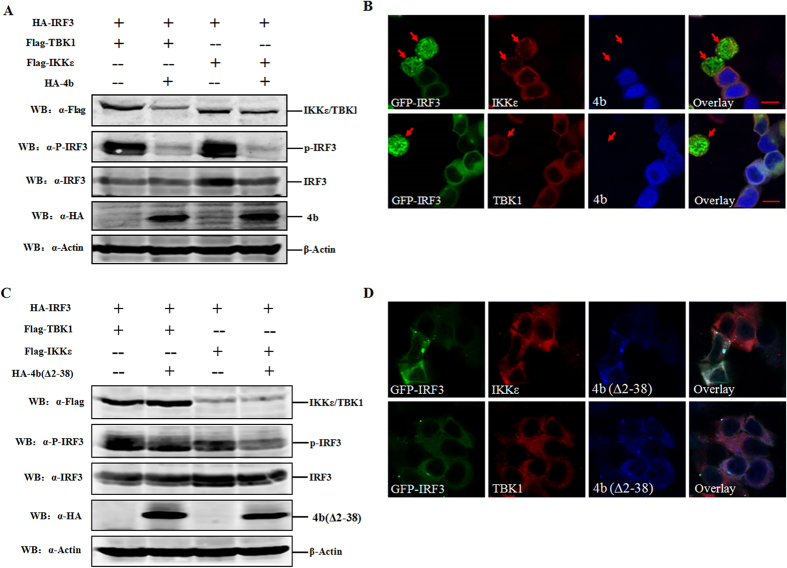
Inhibition of IRF3 phosphorylation and nuclear translocation in cells expressing ORF4b. (**A**) ORF4b inhibits the phosphorylation of IRF3. The 293T cells were co-transfected with different combinations of expression plasmids for HA-tagged ORF4b protein and FLAG-tagged TBK1/IKKε. Cell lysates were subjected to SDS-PAGE for subsequent analysis with anti-FLAG (α-FLAG), anti-HA(α-HA), anti-phosphoIRF3 (α-p-IRF3), anti-IRF3 (α-IRF3), and anti-β-Actin (α-Actin) antibodies. (**B**) ORF4b inhibits nuclear translocation of IRF3. Hela cells were co-transfected with different combinations of expression plasmids for HA-tagged ORF4b protein and FLAG-tagged TBK1/IKKε. Cells were then stained for ORF4b and TBK1/IKKε with anti-HA and anti-FLAG antibodies, respectively. The green (IRF3), red (TBK1/IKKε), and blue (ORF4b) fluorescent signals were merged. Cells were analyzed by confocal microscopy using a 100× objective, and representative images are shown. Scale bar, 20 μm. (**C**,**D**) 4b(Δ2-38) inhibits the phosphorylation and nuclear translocation of IRF3. Experiments were carried out as in Fig. 5AB, except that 4b(Δ2-38) was accessed.

The interaction between ORF4b and IKKε/TBK1, coupled with the results from our previous study showing that SeV-induced nuclear translocation of IRF3 is inhibited by ORF4b protein^15^, suggested that ORF4b may inhibit IKKε/TBK1-induced IRF3 phosphorylation and nuclear translocation. First, the capacity of ORF4b to inhibit IRF3 phosphorylation was tested using a transfection assay. Phosphorylated IRF3 was detected in cultured 293T cells expressing IKKε/TBK1 alone, but with a significant decrease in cells simultaneously expressing IKKε/TBK1 and ORF4b protein, whereas comparable amounts of IRF3 were found in all groups of cells (Fig. 5A). Moreover, ORF4b protein inhibited IKKε/TBK1-induced nuclear translocation of IRF3. Hela cells were co-transfected with IKKε/TBK1 and GFP-IRF3, along with ORF4b-expressing plasmid or an empty vector. At 24 h p.t., cells were fixed and permeabilized, and intracellular staining for IKKε/TBK1 was performed using anti-Flag monoclonal antibody (red) with anti-HA monoclonal antibody staining to visualize ORF4b (blue). Nuclear translocation of GFP-IRF3 was observed (green) in cells expressing IKKε/TBK1, but not ORF4b (Fig. 5B, indicated as red arrows). However, in ORF4b-expressing cells, the translocation of IRF3 was inhibited. These results suggest that IKKε or TBK1 mediated IRF3 phosphorylation, and nuclear translocation was inhibited by ORF4b protein. As ORF4b (Δ2–38) could also interact with IKKε and TBK1, unsurprisingly, it could inhibit IKKε/TBK1-induced IRF3 phosphorylation and nuclear translocation as well (Fig. 5C,D).

### ORF4b protein does not affect the interaction between IKKε/TBK1 and their IRF substrates

**Figure 6:**
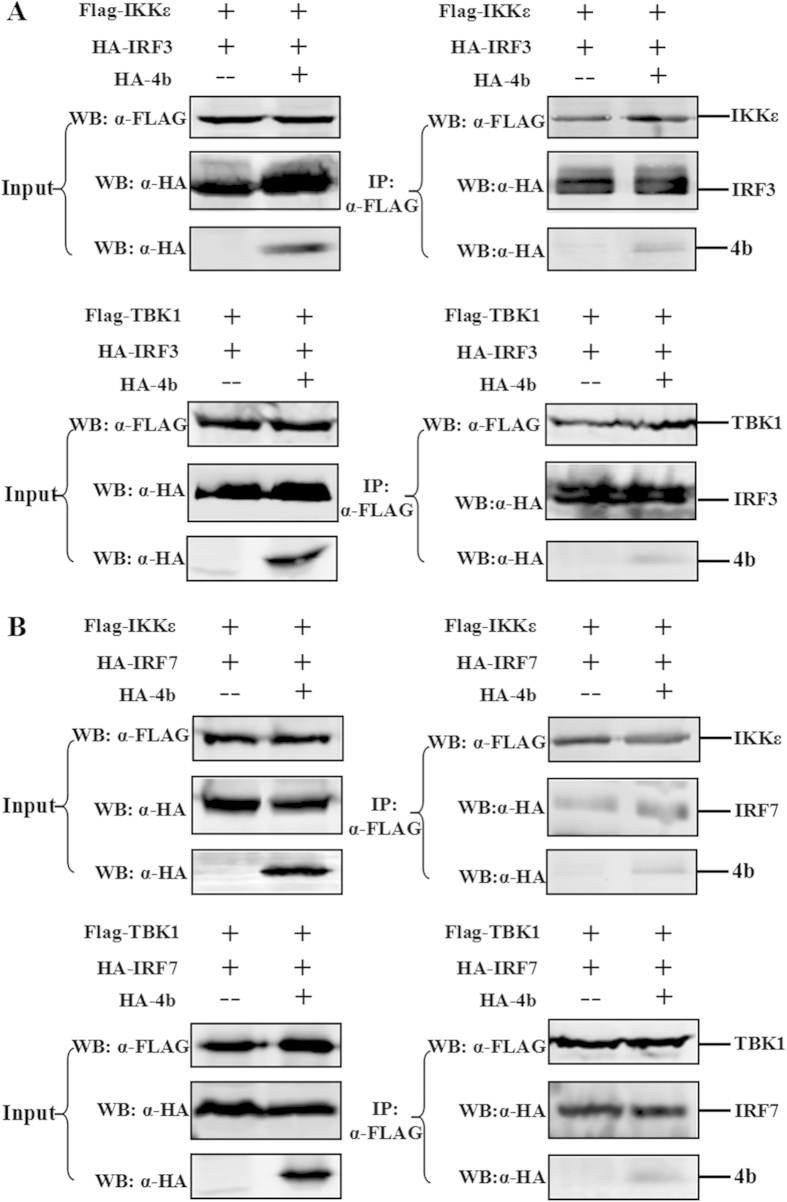
ORF4b does not affect the interaction between IKKε/TBK1 and their IRF substrates. The 293T cells were co-transfected with expression plasmids for FLAG-tagged IKKε/TBK1, plasmids expressing HA-tagged IRF3 (**A**) or HA-tagged IRF7 (**B**), together with a plasmid expressing HA-tagged ORF4b or empty vector. Input cell lysates (left panel) and immunoprecipitates (right panel) were analyzed by Western blotting (WB) with anti-FLAG (α-FLAG) and anti-HA (α-HA) antibodies.

When MAVS receives the activation signals from MDA5/RIG-I, it subsequently recruits and activates TBK1 and IKKε^22^,^23^,^24^. Activation of TBK1 and IKKε results in the phosphorylation of IRF-3 or IRF-7, translocation to the nucleus, and induction of IFN-β mRNA transcription and translation. The finding that ORF4b protein binds IKKε and TBK1, coupled with the inhibition of IRF3 phosphorylation induced by these kinases, suggests that ORF4b may block the interaction between IKKε or TBK-1 and their IRF substrates. To explore this hypothesis, co-immunoprecipitation experiments were performed. First, cells were co-transfected with IKKε/TBK1 and IRF-3 plasmids in the absence or presence of ORF4b-expressing plasmid. IKKε/TBK1 was immunoprecipitated using monoclonal anti-Flag antibody, and co-immunoprecipitation of IRF3 was assessed by Western blotting using an anti-HA monoclonal antibody. The results showed that both IKKε/TBK1 pulled down IRF3, whereas there was no difference between the absence and presence of ORF4b (Fig. 6A). The same result was observed between IKKε/TBK1 and IRF7 (Fig. 6B), which further confirmed that ORF4b does not influence the interaction between IKKε or TBK1 and their IRF substrates.

### ORF4b protein suppresses formation of the IKKε and MAVS complex

**Figure 7:**
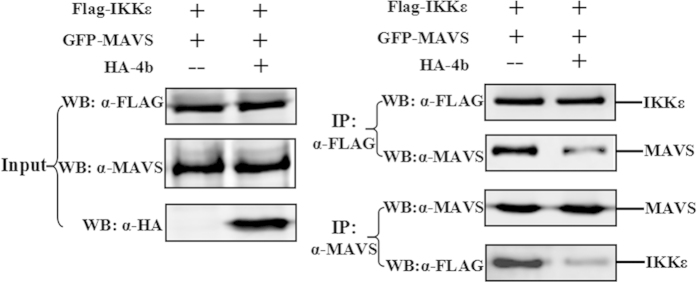
ORF4b suppresses the formation of complex between IKKε and MAVS. The 293T cells were co-transfected with expression plasmid for FLAG-tagged IKKε, plasmid expressing GFP-tagged, together with a plasmid expressing HA-tagged ORF4b or empty vector. Input cell lysates (left panel) and immunoprecipitates (right panel) were analyzed by Western blotting (WB) with anti-FLAG (α-FLAG) and anti-MAVS (α-MAVS) antibodies.

Previous studies have shown that IKKε interacts with MAVS^23^,^29^, and we further explored whether ORF4b protein impairs this process. To this end, 293T cells were co-transfected with IKKε and MAVS, along with ORF4b-expressing plasmid or an empty vector. IKKε and MAVS were immunoprecipitated by adding anti-Flag or anti-MAVS monoclonal antibody to the cell lysates, and co-immunoprecipitation was assessed by Western blotting using both antibodies. As expected, binding of MAVS to IKKε was readily demonstrated in the absence of ORF4b protein, with an obviously decreased interaction observed in the presence of ORF4b protein (Fig. 7). Collectively, these results indicate that ORF4b protein suppresses the IKKε and MAVS complex.

## Discussion

The IFN system plays an important role in the host defense against viral invasion^8^,^9^,^30^. Consequently, to combat the antiviral effects of IFN, many viruses (including coronavirus) have adapted strategies to evade or even inhibit key elements of host IFN responses, and multiple virus-encoded proteins are involved in this process^30^. To the best of our knowledge, at least nine proteins encoded by SARS-CoV have been identified as IFN antagonists: nsp1, PLP, nsp7, nsp15, N, M, ORF3b, ORF6, and ORF9b^31^,^32^,^33^,^34^,^35^,^36^,^37^,^38^,^39^,^40^,^41^. Similar to SARS-CoV, previous studies have shown that MERS-CoV infection failed to elicit strong type I or III IFN or pro-inflammatory innate immune responses in *ex vivo* respiratory tissue cultures^11^,^12^, and the infection is impeded to some extent by exogenously added IFNs^13^,^14^. Until recently, this inhibition was thought to be mediated through viral structural, accessory, and nonstructural proteins M, ORF4a, ORF4b, ORF5, and PLpro^15^,^16^,^17^,^18^,^19^,^20^. However, the underlying molecular mechanisms remained unknown, excluding ORF4a. In a recent study^18^, ORF4a protein was shown to interact with PACT in an RNA-dependent manner and inhibit PACT-induced activation of RIG-I and MDA5.

In our previous study, ORF4b could prevent the activation and nuclear translocation of IRF3 in response to SeV infection^15^. Here, we further investigated the molecular mechanisms by which ORF4b protein inhibits IFN expression signaling. As SeV is a strong inducer of the RLR-mediated IFN signaling pathway^23^, ORF4b-mediated inhibition of SeV-induced IFN-β production, and IRF3 activation suggests that the protein targets one or several components of the RLR signaling pathway. Thus, we screened several components of the RLR signaling pathway and determined ORF4b could specifically bind to MDA5, IKKε, and TBK1. ORF4b protein interacted with both the MDA5C (containing the amino-terminal CARD domain, aa 1–287) and MDA5H (containing the carboxyl terminal helicase domain, aa 287–1025), showing a stronger interaction with MDA5C. Because MDA5 delivers the activation signal to MAVS through a direct interaction via its CARD motif, we explored whether ORF4b protein impaired this process. However, ORF4b did not affect this process, indicating that the interaction between ORF4b and MDA5 may not contribute to ORF4b-mediated inhibition of IFN-β induction by impairing formation of the MDA5 and MAVS complex. In addition, ORF4b specifically reduced IKKε and TBK1-mediated IRF3 phosphorylation and nuclear translocation. RLRs and Toll-like receptors (TLRs) are the two main host PRRs for RNA viruses by recruiting different downstream adaptors for IFN signaling. However, they both utilize IKKε and TBK1 to phosphorylate IRFs, thereby inducing the subsequent transcription and synthesis of IFN-β^8^,^9^. Given the importance of IKKε and TBK1 in the IFN signaling pathway, it is not surprising that viruses have evolved mechanisms that target them to inhibit IFN production. Several other RNA viruses are known to encode proteins that impair IKKε/TBK1 function and antagonize IFN response. The M protein of SARS-CoV^40^, NS2 protein of HCV^42^, VP35 protein of Ebola virus^29^, V proteins of paramyxoviruses^43^, and P proteins of rabies^44^ inhibit the phosphorylation of IRF3 by impeding the formation of TRAF3-TANK-TBK1/IKKε complex by acting as an alternative substrate and/or by disrupting the IKKε/TBK1 interaction with other signaling components including MAVS, IRF3, and IRF7. When we explored how ORF4b leads to the inhibition of IRF3 phosphorylation, we found that ORF4b did not disrupt the IKKε/TBK1-IRF3 or IKKε/TBK1-IRF7 complex, but disturbed IKKε-MAVS complex formation. MAVS is an important adaptor and upstream binding partner of IKKε and TBK-1 that is important for the activation of these kinases and the production of IFN-β in the RLR pathway^22^,^23^,^24^. The capacity of ORF4b to target the IKKε-MAVS interaction suggests that ORF4b may be able to at least partially prevent the activation of IKKε and TBK-1 kinases, serving as one of the mechanisms to inhibit IRF3 phosphorylation^29^. As current opinion comes to that MAVS has to recruit TRAF3, which subsequently activate IKKε and TBK-1, it will be necessary to testify whether ORF4b affects TRAF3 interaction with either MAVS, IKKε or TBK-1 in the future work. And furthermore, it remains unclear whether ORF4b can also inhibit IKKε/TBK1 kinase activity through some other mechanism, such as inhibition of IKKε/TBK1 kinase activity. To clarify these, biophysical methods such as *in vitro* kinase assays could be performed to assess the impact of ORF4b on TBK1 and IKKε activity.

To further confirm the possible inhibition step of ORF4b, we used transducer proteins in the RLR pathway as an IFN inducer, including RIG-I, MDA5, MAVS, IKKε, TBK1, and IRF3. Interestingly, ORF4b not only inhibited the induction of IFN-β by RIG-I, MDA5, MAVS, IKKε, and TBK-1, but also IRF3 and IRF7-induced production of IFN-β. Furthermore, when we abolished nuclear import of ORF4b, it could no longer inhibit IRF3 and IRF7-induced production of IFN-β, RIG-I, MDA5, MAVS, IKKε, or TBK-1, suggesting that ORF4b could inhibit the induction of IFN-β both in the cytoplasm and nucleus. Several viral proteins have been shown to inhibit the transcription of mRNA of IFN-β transcription in the nucleus through different mechanisms. HSV NS1 protein associates with IRF-3 and its transcriptional coactivator CBP, leading to disrupted association of IRF-3 to CBP and reduced binding of IRF-3 to the IFN-β promoter^45^; RVFV NSs protein binds SAP30 (a subunit of complexes intervening in gene transcription regulation) and SAP30 associates with YY1 (the activator/repressor of IFN transcription), forming a multi-protein repression complex on the IFN-β promoter^46^. Viral proteins could also inhibit RNA polymerase II transcription by triggering degradation of RNAP II^47^,^48^,^49^,^50^, triggering the loss of a specific phosphorylated form of RNAP II^51^, associating with MED8 (component of mediator, a protein complex necessary for mRNA production)^52^ and interacting with cyclin-dependent kinase 9 (CDK9) to inhibit RNA Polymerase II transcription elongation^53^. However, fractionation of cytoplasmic and nuclear proteins could be used to detect the mechanisms how ORF4b inhibit IFN production in nucleus and determine if ORF4b utilizes similar mechanisms as those aforementioned.

The NS1 protein of Influenza is one of the most well-understood interferon antagonist among viral proteins, multiple mechanisms for NS1 have been described that form the basis of its antagonist activity, both in the cytoplasm and the nucleus^54^,^55^. For example, in the cytoplasm, NS1 specifically binds to TRIM25 and subsequently inhibits the ubiqitination of RIG-I and further downstream antiviral signaling events including binding to MAVS^56^; in the nucleus, NS1 can inhibit the maturation and export of cellular mRNAs including IFN-β transcripts, hence, block their expression^57^,^58^. As ORF4b share similar properties with NS1, such as subcellular localization and multiple anti-interferon actions^59^, there may be some shared anti IFN mechanisms, especially in the nucleus, which can be referred to in the future work.

## Conclusions

We reported an additional mechanism for MERS-CoV evasion of host IFN responses in which ORF4b specifically binds to TBK1 and IKKε, suppresses the molecular interaction between MAVS and IKKε, and inhibits IRF3 phosphorylation and subsequent activation of IFN-β production signaling. Furthermore, our results suggest that ORF4b can also inhibit the induction of IFN-β in the nucleus, although the underlying mechanism requires further investigation. Compared to our understanding of SARS-CoV, the molecular mechanism by which MERS-CoV regulates IFN activity remains largely unknown. The identification of virus-encoded IFN antagonists and an understanding of the mechanism of action of each antagonist may provide novel therapeutic targets and more effective vaccines.

## Materials and Methods

### Cells and virus

Hela and 293T cells were cultured in Dulbecco’s Modified Eagle Medium (Invitrogen, Carlsbad, CA) supplemented with 10% fetal bovine serum (HyClone, Logan, UT), penicillin (100 U/mL), streptomycin (100 g/mL), nonessential amino acids (0.1 mM), and L-glutamine (2 mM) (Invitrogen, Carlsbad, CA). SeV (Cantell strain) was propagated at 37 °C in 10-day-old embryonated chicken eggs.

### Plasmid constructs

Plasmids of pGL3-IFNβ-luc, pRL-SV40, pEGFP-IRF-3, and pCAGGS were previously described^15^. Plasmids expressing Flag-tagged MDA5C and MDA5H were constructed as previously reported^28^. Plasmids expressing GFP-tagged MAVS, Flag-tagged MDA5, RIG-I, TBK1, and IKKε were previously described^60^,^61^. The plasmid expressing HA-tagged IRF7 was a gift from Dong-Yan Jin (Department of Biochemistry and Microbiology, The University of Hong Kong).

### Western blot analysis

The 293T cells were seeded in 12-well dishes and transfected with the indicated plasmids. At 24 h post-transfection (p.t.), the cells were lysed in ice-cold RIPA buffer (50 mM Tris-HCl (pH 7.5), 150 mM NaCl, 1% Triton X-100, 0.1% SDS, and 0.5% sodium deoxycholate) supplemented with a protease inhibitor mixture (Sigma, St. Louis, MO). The lysates were kept on ice for 10 min, centrifuged, and resolved by 10% SDS-PAGE. The proteins were then transferred to a PVDF membrane (Pall, Port Washington, NY), blocked with 5% skim milk in PBST for 1 h, and probed with the indicated primary antibodies at an appropriate dilution overnight at 4 °C. The following day, the membrane was incubated with the corresponding IRDye 800-labeled IgG secondary antibodies (Li-Cor Inc., Lincoln, NE) and scanned using the Odyssey Infrared Imaging System (Li-Cor Inc., Lincoln, NE).

### Indirect immunofluorescence assay and confocal microscopy

Hela cells were seeded onto glass coverslips in a 24-well plate and transfected with the indicated expression plasmids using the HD transfection reagent (Promega, Madison). At 24 h p.t., the cells were fixed in 4% formaldehyde, permeabilized in 0.5% Triton X-100, blocked in 5% BSA in PBS, and then probed with primary antibodies for 1 h at room temperature. Primary antibodies used were mouse anti-HA and rabbit anti-flag (Sigma-Aldrich, St. Louis, MO). The cells were washed three times with PBS and then incubated with either goat anti-mouse Ig conjugated with Alexa fluor 405 or goat anti-rabbit Ig conjugated with Alexa fluor 594 at a dilution of 1:500 for 1 h (Invitrogen, Carlsbad, CA). The cells were then washed and stained with 4, 6-diamidino-2-phenylindole (DAPI) (Invitrogen, Carlsbad, CA) to detect nuclei. Fluorescence images were obtained and analyzed using an LSM 510 laser-scanning confocal microscope (Carl Zeiss).

### Immunoprecipitation

Transfected cells were lysed in ice-cold RIPA buffer (50 mM Tris-HCl (pH 7.5), 150 mM NaCl, 1% Triton X-100, 0.1% SDS, and 0.5% sodium deoxycholate) supplemented with a protease inhibitor mixture (Sigma, St. Louis, MO). Lysates of cells were incubated overnight at 4 °C with monoclonal antibodies against HA, Flag, and GFP (Sigma-Aldrich, St. Louis, MO) in the presence of protein A/G agarose beads (Santa Cruz Biotechnology, Santa Cruz, CA). Immunocomplexes captured on the protein A/G agarose beads were subjected to electrophoresis and immunoblotting analysis.

### Transfection and reporter gene assays

Reporter assays were performed as previously described^15^. Briefly, 293T cells were seeded in 24-well plates at a cell density of 2.5 × 10^5^ cells per well. The next day, cells were transfected with a control plasmid or plasmids expressing RIG-I, MDA5, MAVS, TBK1, IKKε, and ORF4b, along with pGL3-IFN-β-Luc and pRL-SV40 using HD transfection regents (Promega, Madison). The total amount of DNA was kept constant by adding empty control plasmid. Cells were harvested, lysed, and analyzed with a Dual-Luciferase Reporter Assay System according to the manufacturer’s protocol (Promega, Madison). Values for the samples were normalized using the Renilla luciferase values and expressed as percentages of the value for the negative control.

## Additional Information

**How to cite this article**: Yang, Y. *et al.* Middle East respiratory syndrome coronavirus ORF4b protein inhibits type I interferon production through both cytoplasmic and nuclear targets. *Sci. Rep.* **5**, 17554; doi: 10.1038/srep17554 (2015).
